# Embolization of uterine artery pseudoaneurysm during pregnancy: case report and review of the literature

**DOI:** 10.1515/crpm-2022-0010

**Published:** 2022-12-19

**Authors:** Charlotte K. Metz, Larry Hinkson, Bernhard Gebauer, Wolfgang Henrich

**Affiliations:** Department of Obstetrics, Charité – Universitätsmedizin Berlin, Corporate Member of Freie Universität Berlin and Humboldt-Universität zu Berlin, Berlin, Germany; Department of Radiology, Charité – Universitätsmedizin Berlin, Corporate Member of Freie Universität Berlin and Humboldt-Universität zu Berlin, Berlin, Germany

**Keywords:** embolization, pregnancy, uterine artery pseudoaneurysm

## Abstract

**Objectives:**

Uterine artery pseudoaneurysm (UAP) is a rare but sinister complication during pregnancy. Diagnosis can be made by color Doppler ultrasound. Previous abdominal- and obstetric surgery increase the risk for UAP formation.

**Case presentation:**

We present a case of a 36 year young healthy women, presenting at 27 weeks of gestation with acute lower abdominal pain. UAP was detected by color Doppler ultrasound. An endovascular coil embolization was performed, with good maternal and fetal outcome. Furthermore, a review of the literature looking at UAP embolization in pregnancy was performed.

**Conclusions:**

UAP is reported to appear as a complication of endometriosis. UAP should be treated by endovascular coil embolization, which is a safe and with almost 100% success rate an effective treatment during pregnancy.

## Introduction

The uterine artery pseudoaneurysm (UAP) is a rare but sinister complication during pregnancy and the postpartum period. A postpartum haemorrhage is associated in 0.3–1% of cases with UAP and is a significant cause for maternal and fetal morbidity and mortality [1]. The risk of rupture increases proportional with size and pressure of the UAP [2]. UAP diagnosis can be made by color Doppler sonography, presenting an anechoic or hypoechoic mass with a turbulent arterial linked-flow [2]. Most of UAPs during pregnancy are treated by selective transcatheter coil embolization [3], [4], [5], [6], [7], [8], [9]. Laparotomy with direct internal iliac artery ligation or vascular surgery are further treatment options by UAP related uncontrollable and life-threatening haemorrhage during pregnancy.

## Case presentation

We present the case of a 36 year young patient (Gravida 2, Para 0) presented at 26+2 weeks of gestation with acute onset of left-sided lower abdominal pain. She had one early miscarriage. The patient was healthy without any known pre-existing conditions. She had no previous surgical interventions. There was no history of medications, smoking, alcohol or drugs. The pregnancy was conceived naturally and the second trimester anomaly screening scan was normal.

**Figure 1:**
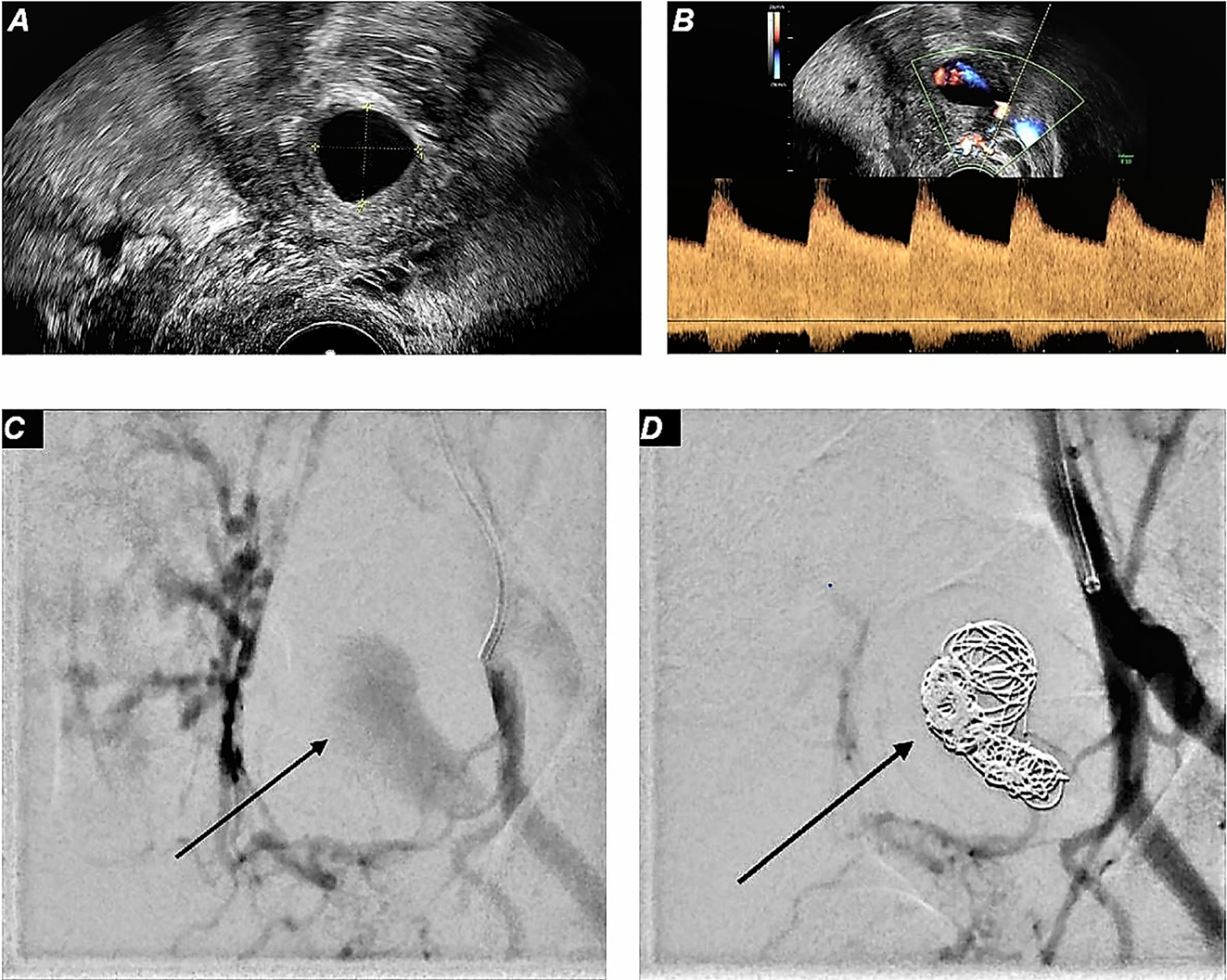
Uterine artery pseudoaneurysm. (A) Transvaginal ultrasound showing an anechoic lesion on the left side, suspicious for uterine artery pseudoaneurysm. (B) Transabdominal color Doppler ultrasound and pulsed wave showing high blood flow in anechoic lesion during systole coming from left uterine artery, suspicious for uterine artery pseudoaneurysm. (C) Digital subtraction angiography image of left uterine artery showing pseudoaneurysm filling with contrast medium. (D) Digital subtraction angiography image of left uterine artery showing occluded pseudoaneurysm after interventional radiological coil embolization.

On admission, the C-reactive protein was elevated at 64.5 mg/L and leukocytes were 16.32/pL. The hemoglobin level was 10.1 g/dL. The cardiotocography (CTG) was constantly physiological. Transvaginal ultrasound was performed and revealed a 2 × 2 cm anechoic structure arising from the left uterine artery. High turbulent arterial flow could be seen with color Doppler ultrasound. The findings were suspicious for a pseudoaneurysm of the left uterine artery (Figure 1A and B).

As incidental finding a solid mass 6 × 3 × 3 cm was seen in the rectovaginal septum with increased perfusion. A differential diagnosis of asymptomatic intestinal endometriosis was considered. Fetal sonography showed grow appropriate for gestational age and organ screening was unremarkable. Uterine artery perfusion was normal. Magnet Resonance Imaging (MRI) was performed to confirm the diagnosis. Following a multidisciplinary review of the clinical symptoms and the diagnostic findings on ultrasound and MRI (which included obstetricians, interventional radiologists and gynaecologists), UAP was seen as the most reasonable diagnosis.

Antibiotic therapy was started with Cefuroxime intravenously and a corticosteroid prophylaxis was given. The risk of spontaneous UAP rupture with maternal haemorrhage and fetal complications were balanced with the risk of an interventional embolization and radiation exposure during pregnancy. In view of the high probability for vessel occlusion, prolongation of the pregnancy and low intervention risks a catheter guided selective coil embolization was chosen. The patient provided inform consent.

The procedure was performed through the left femoral communis artery approach and the UAP was embolized with seven 0.018” coils, until a vascular stasis could be seen. Post procedure angiographic control showed a minimal rest-perfusion in the UAP. Following this, multiple attempts to insert an additional stent graft (Papyrus 3.5 mm/20 mm) via a 0.014” microwire in the region of the vascular defect failed due to prior stenosis. After removal of the non-deployed Stentgraft and all wires no rest-perfusion was detected in the UAP, presumably caused by vascular spasm or delayed thrombogenic effect of coils (Figure 1B and C). Thereafter, post interventional angiography showed total occlusion of the UAP with good perfusion of left distal uterine artery and placenta. Total radiation exposure was 2,100.5 μGym^2^ with a fluoroscopy time of 16 min. The embolization procedure was otherwise.

The further course of the pregnancy was uneventful. The patient continued to receive further regular prenatal care and the UAP remained occluded. Because of the ultrasound and MRI findings, suspicious for endometriosis, patient was transferred to our endometriosis centre. A rectovaginal endometriosis with colon infiltration was confirmed, without the need of intervention. Based on the endometriosis findings and breech presentation the indication for elective caesarean at 39+0 weeks of gestation was made. Intraoperatively, adhesion of the left fallopian tube with the uterus was found. The rectovaginal endometriosis could not be palpated from intraabdominal. A male baby was born, 3,620 g with APGAR Scores of 9 at 1 min and 10 at 5 min and an arterial pH of 7.28. Postpartum, there were no complications, mother and child were doing well. Two years later, UAP showed still occlusion in transabdominal ultrasound control consultation.

## Discussion – review of the literature

To our knowledge this is the first literature review of uterine artery pseudoaneurysm detected and managed prenatally. We performed a review of the literature looking at UAP embolization in pregnancy. The database PubMed was reviewed over the period 1997 to present to identify relevant case reports of uterine artery pseudoaneurysm found prenatally and the management with embolization.

The following key terms were used to search the relevant literature: (1) Uterine artery pseudoaneurysm and pregnancy and embolization, (2) UAP/Pregnancy/Embolization. No filters or limitations were used. Additionally we screened the references of corresponding case reports to search for further relevant articles.

**Figure 2:**
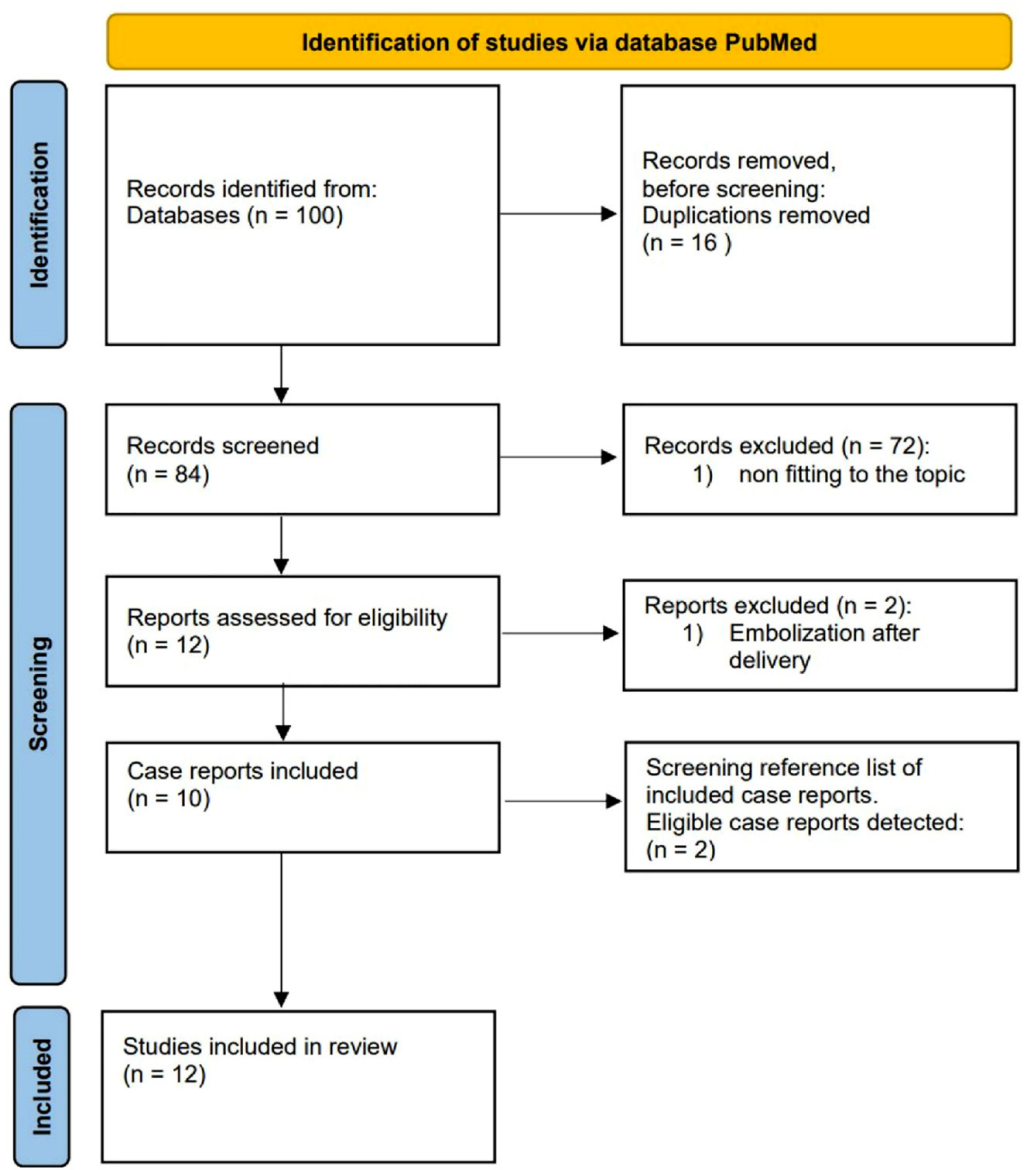
Searching algorithm PubMed: selection of case reports for inclusion [10].

Finally, 12 case reports were included for review in our manuscript (Figure 2).

All 13 case reports, including 12 case reports detected via PubMed and our presented case report will be discussed in the following.

UAP rupture can result in life-threatening haemodynamic instability with increased maternal and fetal morbidity and mortality. Pregnancy-associated hormonal changes can increase the risk for UAP appearance and rupture [11]. These hormonal changes supposably cause arterial intima and media hypoplasia and membrane permeability alterations [11]. Additionally, hemodynamic blood pressure changes in pregnancy, shown by a general increase in blood flow with the lowest peripheral vascular resistance in the second trimester and the greatest increase of maternal heart rate in the third trimester. On analysing the case reports, UAPs occurred associated with hemodynamic changes, either to 53.9% in second trimester (7/13 women) [3, 6, 9, 12], [13], [14], [15] or with 46.2% in third trimester of pregnancy (6/13 women) [4, 5, 7, 8, 16] (Table 1).

**Table 1:** Case reports: embolization of uterine artery pseudoaneurysms during pregnancy.

Case reports	Roeckner et al. [12]	Van Coppenolle et al. [4]	Cornette et al. [16]	Gavanier et al. [3]	Ugwumadu et al. [5]	Konishi et al. [6]	Laubach et el. [7]	Maignien et al. [8]	Mulkers et al. [13]	Zilberman et al. [9]	Zwimpfer et al. [15]	Wallach et al. [14]
Age, years	29	27	37	36	38	30	33	29	35	30	34	29
Week of gestation	15	29	27	20	26	24	28	27	18	14	23	16
Gravidity	I^a^	I	II	II	I	III^b^	I	II	I	I	I	I
Nullipara	X	X	–	–	X	X	X	X	X	X	X	X
IVF	–	–	–	–	–	–	X	X	X	–	–	–
Surgery history	–	–	Laparoscopy	C-section Adnexectomy	–	–	2x Laparoscopy	2x Laparotomy	Laparoscopy	–	Cervical conization	–
Endometriosis	–	–	–	X	–	–	X	X	–	X	X	–
Symptoms												
Painless	X											
Painless vaginal bleeding			X				X			X		X
Abdominal pain		X				X			X	X	X	X
Pain in iliac fossa				X	X			X				
Hemodynamic situation				Tachycardia		Tachycardia, hypotension						
Side-symptoms	–	–	Dyschezia	Pale	Nausea, Vomiting, diarrhoea	Anaemia, Ascites	–	–	Nausea, Vomiting, Diarrhoea, Urge to move	–	Dyschezia, Brown vaginal discharge	–
Intervention												
Trans-abdominal/transvaginal ultrasound	X	X	X	–	X	X	X	X	X	X	X	X
MRI	X^d^	–	X^d^	–	X^c^	–	–	–	X^c^	–	X^c^	X^c^
CT	–	X^d^	–	X^d^	–	X^d^	–	–	–	–	–	–
MRA	–	–	–	–	–	–	X	X	–	X	–	–
DSA	–	–	–	–	–	–	X	–	X	–	–	–
Laparoscopy	–	–	–	–	–	–	–	–	X	–	–	–
Differential diagnosis	Arterio-venous malformation	–	Endometriosis	–	–	Splenic artery aneurysm	–	–	Ovarien-(sub)torsion	–	Endometriosis	–
I. Treatment	Thrombin injection	Coiling	Mixture or NBCA and ethiodized poppy seed oil	Coiling	Coiling	Coiling	Coiling	Coiling	Microsphere particles + gelatine	Coiling	Mixture or NBCA and ethiodized poppy seed oil	Thrombin injection
Insufficient	X	–	X	–	–	–	–	–	X	–	–	X
Rupture during treatment	–	–	–	–	–	X	–	–	–	–	–	–
II. Treatment	Coiling	–	Mixture or NBCA and ethiodized poppy seed oil + coiling	–	–	2x Mixture of NBCA and ethiodized poppy seed oil + coiling	–	–	Coiling at 30 weeks: Recurrence of UAP -> Glubran injection	–	–	Coiling
Occlusion of UAP	X	X	X	X	X	X	X	X	X	X	X	X
Mode of delivery	Forceps-assisted vaginal delivery	C-section	C-section	C-section	C-section	Vaginal birth	C-section	C-section	C-section	C-setion	C-section	Forceps-assisted vaginal delivery
	37+0	37+2	37+5	34+2	38+0	41+3	30+4	31+1	34	38	37	37+0
Neonatal parameters	XY, 2,780 g Apgar: 8/9	2,150 g Good adaptation	XX, 2,820 g, healthy	XX, 2,600 g, healthy	XY, 2,696 g healthy	XX, healthy	XX, 1,660 g, Apgar: 3/5/8 pH 7.0	XX, 1,420 g Apgar: 8 pH 7.18	XY, 2,065 g, Apgar: 8/9 healthy	XY, 2,900 g, healthy	XX, 2,670 g, Apgar: 6/7/8 pH 7.31	Healthy
Postpartum no-UAP related complication	X	X	X	–	X	X	Suspected for placental abruption	X	X	X	X	X

^a^No previous pregnancies named; ^b^In case report named as Gravida II, without counting current pregnancy; ^c^Without contrast medium; ^d^With contrast medium.

Beside pregnancy, vascular diseases, arterial degeneration processes, inflammation and trauma are additional risk factors for an arterial pseudoaneurysm formation and its rupture. Furthermore, previous abdominal surgery in particular previous caesareans are considered as risk factors for UAP occurrence [17]. Prior traumatic deliveries, manual placenta removal, forceps delivery, vacuum extraction, termination of pregnancy and evacuation of the uterus increase the risk for UAP [17]. During surgical interventions especially in the low abdominal quadrants, uterine arteries can be lacerated or injured, which support the development of a pseudoaneurysm. Post surgical adhesion may create traction on surrounded structures and vessels which modulate anatomic tissue relation ships [17]. Concerning the reported case reports, previous abdominal surgery was reported by 46.2% of the women (6/13 women), including three laparoscopies, one double laparotomy, one adnexectomy and one cervical conization [3, 7, 8, 13, 15, 16] (Table 1). In the obstetric history prior miscarriages were reported in 23.1% of cases (3/13 women) [6, 8] (Table 1). Interestingly, 84.6% (11/13 women) of pregnant women with UAP occurrence were nullipara of whom 72.7% (8/11) were primigravida [4], [5], [6], [7], [8], [9, 12], [13], [14], [15] (Table 1).

Additionally, nearly half of the women, 46.2% (6/13 women), showed endometriosis as an additional diagnosis [3, 7], [8], [9, 15] (Table 1). Based on the current literature, endometriosis is not reported as a general risk factor for UAP occurrence, although UAP has already been reported as a rare complication of endometriosis [18]. High pregnancy-related progesterone level are usually associated with regression of endometriosis symptoms and progression, except when caused by the decidualisation process, which can support new vascular changes. Changes of permeability, angiogenesis and vascular remodelling induced by intrusion of decidualized endometriotic tissue into the vessel wall can result in vessel dysfunction [18]. Apart from that endometriosis can cause a chronic inflammation, which is related to tissue and vessel fragility resulting in fibrosis and tissue remodelling [18]. Furthermore, endometriotic cells can produce oxidative stress by accumulating free radicals, which could damage the vascular endothelial tissue [18].

The invasive and progressive growth of endometriosis and the systemic inflammatory influence on the vessel function could be an explanation for the high UAP occurrence among women with an endometriosis.

The clinical presentation of UAP is variable. Abdominal pain, pain in the iliac fossa, painless vaginal bleeding can be the symptoms [3], [4], [5], [6], [7], [8], [9, 12], [13], [14], [15] (Table 1). Nevertheless, also a case without pain had been described [12]. Fetal distress in a rare finding. Abdominal pain was the most reported symptom in 53.8% of cases (7/13 women) [4, 6, 9, 13], [14], [15], followed by painless vaginal bleeding in 30.7% (4/13 women) and pain in the iliac fossa in 23.1% of women [3, 5, 7, 8, 14, 16] (Table 1). The gold standard for UAP diagnosis is an initial transabdominal/transvaginal or color Doppler ultrasound. An anechoic or hypoechoic mass with a turbulent arterial blood flow during systole is the typical sonographic presentation [2]. UAPs can be confirmed by angiography, MRI or computer tomography (CT). In 92.3% of cases (12/13 case reports), an initial ultrasound was performed, where an anechoic, echogenic or echolucent mass connected to the uterine artery, mostly with turbulent flow was detected [4], [5], [6], [7], [8], [9, 12], [13], [14], [15], [16] (Table 1). UAPs were confirmed in 46.2% (6/13 cases) by MRI [5, 9, 12, 13, 15, 16] (Table 1). Whereas, in 30.8% of cases (4/13 cases) UAP were diagnosed with magnet resonance angiography (MRA) and/or digital-subtraction angiography (DSA) [7], [8], [9, 13] (Table 1). Three case reports (23.1%), performed a CT for UAP diagnostic because of persistent pain, falling haemoglobin and hemodynamic instability [3, 4, 6] (Table 1).

Differential diagnosis like arterio-venous malformation, splenic artery aneurysm, ovarian torsion, ovarian bleeding and endometriosis should also be considered [6, 12, 13, 15, 16] (Table 1). Arterial embolization during pregnancy is a safe and efficient treatment for UAP occlusion with good clinical outcome and a success rate of nearly 100% [3], [4], [5], [6, 8, 9, 12], [13], [14], [15], [16] (Table 1). Embolization is a minimal invasive intervention with good toleration by both mother and fetus. UAP embolization can prevent life-threatening haemorrhage which might require laparotomy or an emergency caesarean with hysterectomy. Total occlusion of UAP was achieved in all reported case reports with a good fetal and maternal outcome. No fetal or maternal complications, related to the UAP embolization were reported (Table 1). The reported case reports performed to 61.5% (8/13 cases) an endovascular coiling embolization during pregnancy as the primary therapy [3], [4], [5], [6], [7], [8], [9] (Table 1). Konishi et al. was the only case report, where a rupture of UAP occurred during embolization procedure [6]. An additional placement of coils and a double injection of a N-butyl cyanoacrylate solution (NBCA) was needed for total UAP occlusion [6] (Table 1). Two case reports, 15.4%, reported as primary therapy, a percutaneous thrombin injection for UAP embolization [12, 14]. In both patients, embolization was insufficient and an additional coil embolization was required [12, 14]. Percutaneous thrombin injection has the advantage of short intervention time, however higher recanalization rates and thromboembolic complications are reported [19] (Table 1). The remaining three case reports, performed UAP embolization either with a NBCA (e.g. Histoacryl) mixed with ethiodized poppy seed oil (e.g. Lipiodol) or with microsphere particles [13, 15, 16] (Table 1). Cornette et al., used a NBCA solution for embolization. After three days UAP recurrence was detected, which was treated by an additional NBCA injection and coil embolization [16]. Mulkers et al., performed a microsphere particles and absorbable gelatin sponge (e.g. Gelfoam) embolization, which needed additionally coil and glue embolization [13]. Mulkers et al. and Cornette et al. were with 15.4% the only two case reports which reported UAP recurrence after primary UAP occlusion [13, 16]. Zwipfler et al., achieved UAP occlusion by single injection of NBCA (Histoacryl) mixed with ethiodized oil (Lipiodol) in a 1:3 mixture [15]. Arterial coil embolization needed in most of the cases no additional treatment for UAP occlusion in comparison to other embolization methods. UAP rupture during embolization is a rare complication which might require further embolization methods. UAP recurrence after primary occlusion was reported in 15.4% (2/13 cases). When coiling embolization for UAP occlusion was chosen as primary therapy, no insufficiency and UAP recurrence was reported. Endovascular embolization is performed under angiography which including radiation exposure. Radiation exposure during pregnancy should not exceed radiation dose of 0.5 Gy or 50 rad, in order to minimize fetal and pregnancy complications [20]. But the fetus is generally more resistant to radiation during the second and third trimester. In Germany the threshold dose for brain or other organ dysplasia of the fetus after the 10th week is assumed to be approximately 300 mSv, so the dose for an unborn child in Germany should be below 50 mSv (42). Concerning the mode of delivery, patients delivered in 76.9% (10/13 cases) by caesareans [3], [4], [5, 7], [8], [9, 13, 15, 16] (Table 1). Two women (15.4%, 2/13 women) underwent forceps assisted delivery [12, 14] and one women (7.7%, 1/13 women) had a vaginal delivery [6] (Table 1). In the current literature, UAP is not an absolute indication to perform a elective caesarean. A vaginal delivery can be considered after successful UAP occlusion. In conclusion, our data show that UAP is a rare diagnosis during pregnancy however, UAP should be considered as a cause for an acute abdomen or vaginal bleeding even in healthy, pregnant women. Prior abdominal and obstetric surgery are associated to higher the risk of UAP formation. In women with endometriosis, UAP is a possible and not rare reported association. A color Doppler ultrasound can lead to UAP diagnosis by illustrating an anechoic or echogenic mass near the uterine artery with turbulent flow during systole. Endovascular embolization during pregnancy is an effective treatment of the arterial pseudoaneurysm, with low maternal and fetal risk. Endovascular embolization achieved in almost 100% a total UAP occlusion.
